# Standard vs. Calorie-Dense Immune Nutrition in Haemodynamically Compromised Cardiac Patients: A Prospective Randomized Controlled Pilot Study

**DOI:** 10.3390/nu9111264

**Published:** 2017-11-20

**Authors:** Sergey Efremov, Vladimir Lomivorotov, Christian Stoppe, Anna Shilova, Vladimir Shmyrev, Michail Deryagin, Alexander Karaskov

**Affiliations:** 1 Department of Anesthesiology and Intensive Care, E. Meshalkin National Medical Research Center, 630055 Novosibirsk, Russia; vv_lomivorotov@meshalkin.ru (V.L.); v_shmyrev@meshalkin.ru (V.S.); mderyagin@mail.ru (M.D.); mail@meshalkin.ru (A.K.); 2 Department of Intensive Care Medicine, University Hospital, RWTH Aachen University, 52074 Aachen, Germany; christian.stoppe@gmail.com; 3 Laboratory Diagnostics Department, E. Meshalkin National Medical Research Center, 630055 Novosibirsk, Russia; a_shilova@meshalkin.ru

**Keywords:** early enteral nutrition, critical care, cardiac surgery, protein-rich formula, energy-dense formula, enteral energy delivery

## Abstract

Background. The aim of study was to test the hypothesis that early enteral nutrition (EN) with calorie-dense and protein rich enteral formula improves enteral energy and protein delivery in critically ill cardiac patients. Methods. Prospective randomized pilot study of 40 ventilated adult patients undergoing elective cardiac surgery with use of cardiopulmonary bypass receiving inotropic support postoperatively. Patients were to receive either standard isocaloric (1000 Kcal/L and 38 g/L protein) early EN (*n* = 20) or calorie-dense and protein-rich (1300 Kcal/L and 66.7 g/L protein) early EN (*n* = 20). Results. The mean time to EN initiation was 27 ± 11 h. Early EN with the calorie-dense formula provided significantly more energy and protein enteral delivery on the 2nd, (*p* < 0.0001), 5th (*p* = 0.036), and 7th days (*p* = 0.024), and was associated with higher levels of prealbumin concentration on the 14th day (0.13 ± 0.01 g/L and 0.21 ± 0.1 g/L; *p* = 0.04) and significantly increased levels of transferrin on the 3rd, 5th, and 7th day (*p* < 0.05) after surgery. Conclusion. Present findings support hypothesis that early EN using a calorie-dense and protein rich formula leads to better enteral energy and protein delivery and higher levels of short-lived serum proteins.

## 1. Introduction

The impetuous development of malnutrition resulting from increased catabolic requirements as consequence of critical illness is a well-known clinical problem and of special relevance for patients undergoing cardiac surgery [1,2,3,4]. Adequate energy and protein provision and early enteral nutrition (EN) in critically ill patients improve clinical outcomes [5,6,7], decrease hospital costs [8], and are recommended by International guidelines [9,10]. However, a recently published meta-analysis indicates the risk of bias and need to further investigate the potential benefits of EN [11].

Haemodynamic compromise is a well-known obstacle that inhibits the early initiation of EN owing to the risk of mesenteric ischemia. This is of particular relevance for patients after cardiac surgery with an adverse postoperative course. Despite the lack of high quality evidence indicating that early EN mitigate morbidity and mortality [11], recent studies suggest that early EN is well tolerated and safe in patients with vasopressor or inotropic support if the dosage and hemodynamic status of the patient are stable and are provided with careful abdominal and energy monitoring [12,13]. Therefore, nutritional support for patients on moderate to high vasopressor support must be improved [14] to enhance enteral protein–energy provision.

There exist several commercially available enteral foods for nutrition support. As cardiac surgery affects multiple systems, it is obviously challenging to identify an adequate nutrition therapy that provides beneficial affects to all organs. Despite a lack of scientific evidence and specific recommendations for these cohort of critically ill patients, calorie-dense and protein rich feeding may reduce the potential for fluid loading in cardiac surgery patients, representing an attractive option for an intense nutrition therapy. In addition, immune-modulating properties may be of clinical significance in these patients, which are frequently prone to a systemic inflammatory response syndrome, which often result in the development of organ dysfunctions.

Nowadays, data on the effects of an intense caloric-dense, protein rich nutrition therapy with immune-modulating properties in cardiac surgery are sparse, and only few data about effects of enteral nutrition in these patients exist [15]. Therefore this pilot study tested the hypothesis that early concentrated EN improves enteral energy and protein delivery in critically ill cardiac patients.

## 2. Materials and Methods

### 2.1. Protocol Design

**Figure 1:**
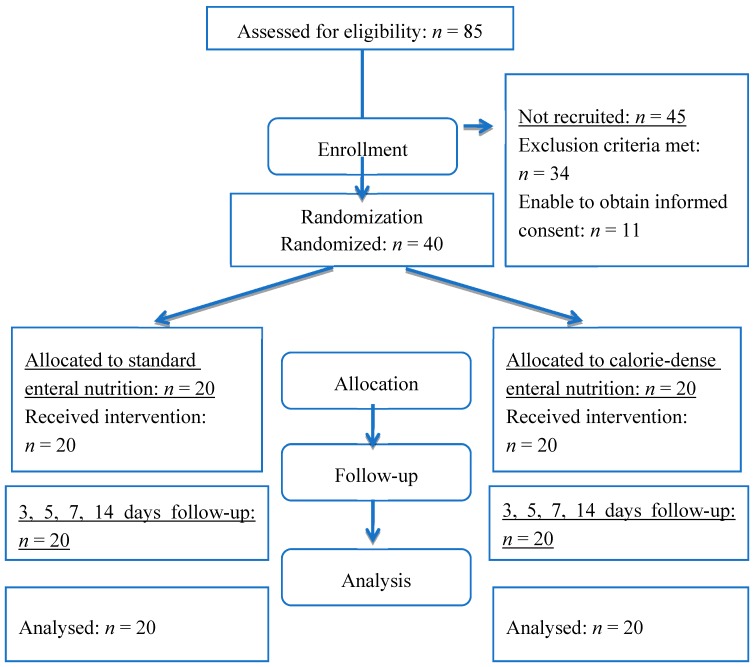
Flow diagram.

This pilot prospective randomised open label study was approved by the local ethical committee of our hospital. From January 2011 to October 2013, cardiac patients were randomised to receive either standard isocaloric isonitrogenic early EN (standard group, *n* = 20; Nutricomp standard liquid, B. Braun Melsungen AG, Melsungen, Germany) or calorie-dense enriched with glutamine and omega-3 polyunsaturated fatty acids early EN (calorie-dense group, *n* = 20; Nutricomp immune liquid, B. Braun Melsungen AG, Melsungen, Germany). The details of the EN formulas are presented in Table 1. Patients were assessed for eligibility during the first 24 h postoperatively. Simple randomisation sequence was electronically generated. Patients were allocated to the intervention using numbered opaque sealed envelopes. Actual assignment of allocated intervention was performed immediately after opening the envelope. The recruitment process is outlined in Figure 1.

### 2.2. Patient Eligibility

The inclusion criteria were as follows: (1) signed informed consent from the patient or their next of kin; (2) age 18 years or older; (3) cardiopulmonary bypass surgery no more than 24 h before eligibility assessment; (4) acute heart failure syndrome; and (5) anticipated time of ventilation more than 48 h. Patients were included only if all listed criteria were met. Acute heart failure syndrome was defined as a vasoactive-inotropic score (VIS) > 5 calculated as follows: VIS = dobutamine (μg·kg^−1^·min^−1^) + dopamine (μg·kg^−1^·min^−1^) + 100 × epinephrine (μg·kg^−1^·min^−1^) + 100 × norepinephrine (μg·kg^−1^·min^−1^) [16].

The exclusion criteria were as follows: (1) acidosis (pH <7.350 and/or serum lactate >4 mM); (2) hypoxia (arterial PaO_2_ >60 mmHg); (3) bleeding; (4) cerebrovascular accident; (5) ileus; (6) diarrhoea (≥3 loose or liquid stools per day); (7) signs of mesenteric ischaemia; and (8) refractory arterial hypotension. Mesenteric ischemia was suspected based on combination of clinical signs (high residual gastric volume, abdominal pain, bloating, ileus), laboratory data (blood lactate, acidosis, leucocytosis), and radiologic symptoms (enlargement and thickening of intestinal loops, pneumotosis). Mesenteric angiography was envisaged for exclusion of occlusive mesenteric ischemia.

### 2.3. Nutritional Intervention

As soon as nutritional support was considered possible (with accordance to inclusion and exclusion criteria), EN was initiated no later than 48 h after surgery via a nasogastric tube at 25 mL/h. Tolerance to EN was assessed according to residual gastric volume measured every 4–6 h. Signs of mesenteric ischemia were also assessed. In cases exhibiting good tolerability, the rate of EN was increased 50% every 12–24 h. Meanwhile, in cases exhibiting insufficient tolerability (residual gastric volume >300 mL), metoclopramide and erythromycin were administered. Parenteral nutrition was started if enterally administered nutrients did not cover 60% of resting energy expenditure (REE) by 74 h after surgery. For parenteral nutrition, three chamber bags containing LCT/MCT lipid emulsion and 70 g amino acids per liter (Nutriflex 70/180 lipid, B. Braun Melsungen AG, Melsungen, Germany) was used. In the cases where patients were receiving nutritional support orally, and this support was found to be suboptimal, EN was continued until the recovery of normal eating habits.

The daily energy target was set using REE, as measured by indirect calorimetry (CCM Express, Medgraphics, St. Paul, MN, USA). Measurements were performed in compliance with the following rules: (1) resting in supine position for more than 30 min before the measurement; (2) at least 12 h of stable rate of nutrient delivery before measurement if the patient received continuous feeding (enteral or parenteral nutrition); (3) constant fraction of inspired oxygen (FiO_2_) less then 0.6 and stable ventilator settings; (4) no general anesthesia during 8 h before the measurement; (5) adequate analgesia; (6) no painful procedures like chest tube removal for at least 1 h before the study; (7) at least 4 h after intermittent hemodialysis; (8) no routine procedures involving healthcare professionals and nursing care during the measurement; and (9) the measurement lasted 15–30 min.

### 2.4. Outcomes

The primary endpoint of this pilot trial was the total daily energy and protein delivery. Serum prealbumin, transferrin, C-reactive protein, blood lactate, VIS, oxygenation index, ventilation time, REE, intensive care unit stay, hospitalisation, and hospital mortality were also analysed.

Prealbumin, transferrin, and C-reactive protein were assessed at baseline and 3, 5, 7, and 14 days after surgery using Thermo Fisher Scientific reagents (Finland) on a Konelab 60 Prime automatic analyser (Thermo Fisher Scientific, Waltham, MA, USA).

Blood lactate and clinical characteristics were assessed daily between the 1st and 7th and at the 14th day after surgery. Lactate was assessed by an automatic blood gas analyzer (Rapidlab 850, Siemens, Munich, Germany).

### 2.5. Statistical Analysis

In this exploratory pilot study, quantitative data are presented as median (interquartile range). Frequencies are presented as the number (%) within the relevant category. Between-group and within-group comparisons were made using the mixed-effects model (with group as fixed and time and patient as random effects) with incorporated correction for multiplicity (Tukey’s method). Based on Levene’s test, the assumption of sphericity was considered as valid. Qualitative characteristics were compared using the χ^2^ test or Fisher’s exact test where appropriate. The level of significance was set at *p* < 0.05 (two-tailed). MedCalc Statistical Software version 13.1.0 (MedCalc Software, Ostend, Belgium) and R (R Development Core Team (2008). R: A language and environment for statistical computing. R Foundation for Statistical Computing, Vienna, Austria) were used for all of the statistical analyses.

## 3. Results

### 3.1. Clinical Characteristics

Baseline characteristics are presented in Table 2. The mean time to EN initiation was 27 ± 11 h.

Regarding hospital mortality, four (20%) and six (30%) patients in the standard and calorie-dense groups died, respectively. The mean ventilation times did not differ significantly and were 5.25 (3.4–6.37) in the standard and 4.75 (3–11.4) days in the calorie-dense group, respectively. The mean intensive care unit stay was 9 (7–11) and 11 (7–23) days in the standard and calorie-dense groups, respectively, and without significant differences; the mean hospitalization duration was 26 (19–21) and 30 (25–33) days, respectively, without significant differences. The characteristics of clinical course of the studied patients are presented in Table 3. There were no significant differences between groups with respect to any of the studied variables. EN was well tolerated by most of the patients; high residual gastric volume was detected in one patient receiving calorie dense nutrition. There were no other complications associated with EN.

### 3.2. Efficacy and Safety of Nutritional Support

Early EN with the calorie-dense formula provided significantly more energy and protein enterally on the 2nd, (*p* < 0.0001), 5th (*p* = 0.036), and 7th days (*p* = 0.024) after surgery, and was significantly associated with higher total energy delivery on the 2nd and 7th days (Table 4). Patients fed the with calorie-dense formula needed less parenteral nutrition. Thus, 16 (80%) and six (30%) patients received parenteral nutrition in standard and calorie-dense groups, respectively (*p* = 0.002). No significant difference was found regarding the extent of organ dysfunctions, as measured by APACHE II Score or SOFA score. Furthermore safety variables, such as number of organ dysfunctions (adverse events), use of vasoactive support or lactate levels did not differ significantly between both treatment groups (Table 3). No differences were found regarding the number of infectious complications.

### 3.3. Laboratory Markers

The dynamics of laboratory nutritional markers (i.e., prealbumin and transferrin) and C-reactive protein as an inflammatory response marker are presented in Table 5. Significant intergroup differences of prealbumin concentration were found on the 14th day after surgery (*p* = 0.04). Transferrin was significantly higher in the calorie-dense group on the 3rd day (*p* = 0.037), 5th day (*p* = 0.011), and 7th day (*p* = 0.018) after surgery. There were no significant intergroup differences with respect to C-reactive protein. 

## 4. Discussion

The present study is the first randomised trial investigating the effects of early initiated calorie-dense, immune-modulating EN support in cardiac surgery patients with acute heart failure syndrome. Previous studies on this topic are observational and merely investigated the feasibility of early EN in this problematic patient group [12,13,17]. The present results are in accordance with those of the abovementioned studies, corroborating the safety of early EN in patients with haemodynamic failure. However, we support restricted opportunities to cover daily nutrient requirements with EN alone.

The ESPEN guidelines for the use of EN in patients with chronic heart failure state that fluid overload should be avoided [18]. Current recommendations in conjunction with known restricted functional capacity of the gastrointestinal tract [19] make calorie-dense feeding an attractive option, especially for cardiac surgery patients with voulme restrictions and inotropic support during the early postoperative course. 

In the present study, calorie-dense feeding was significantly associated with increased levels of serum prealbumin and transferrin during the early post-operative course. These findings can be explained by the fact that patients received more energy and protein for an equivalent amount of delivered EN, resulting in a reduced decrease of transferrin levels measured at the 3rd, 5th, and 7th postoperative day. Yet, we acknowledge that a preoperative assessment of nutrition markers and proteins may have been useful to already identify preoperatively differences between the groups and should be considered in future studies. In addition, it should be noted that despite nutritional support, serum prealbumin and transferrin levels remain below the normal values throughout the study. These findings may indicate that nutrition support was still inadequate due to impaired metabolism or may demonstrate the slow response of these nutrition markers, although previous studies revealed prealbumin as a sensitive marker for diagnosis of malnutrition and for the assessment of postoperative nutritional support [20,21]. However, taking into account multifactorial causes of lowered serum protein levels during critical illness, (catabolism, reprioritisation of protein synthesis by the liver, and derangements of vascular permeability [22,23]) any serum protein let doubt its role as a valid nutritional marker in the setting of critical illness. Thus, increasing the levels of short lived proteins may reflect the overall recovery and decline of systemic inflammation, as reflected by the dynamics of CRP.

Furthermore, almost half of the patients in the present study received renal replacement therapy, which is associated with an additional loss of amino acids, including glutamine via the dialysis membrane [24]. Although nutritional needs will be met more quickly with a calorie-dense formula, our results demonstrated that the studied enteral formulas have a similar effectiveness, in terms of adverse effects associated with EN.

Besides increased caloric content, the calorie-dense formula used in the present study also contained fibre, high concentrations of micronutrients, and glutamine. Glutamine, which is the most abundant amino acid in the human body, has various non-nutritive effects [25]. However, its clinical effects remain controversial and vary depending on the mode of administration and dosage [26]. Thus, recently REDOX trial shows safety concerns of glutamine in high doses among critically ill patients [27].

This pilot study supports the feasibility of early initiation and acceptable tolerance to energy-dense hypernitrigenic EN among hemodynamically compromised cardiac surgery patients. Its results may be used for planning future studies investigating different protein doses or immune modulating effects of EN as these topics are among the top nutritional questions that need to be systematically addressed in the near future [3].

### Limitations

The main limitation of the present study is the small number of subjects; thus, the benefits of calorie-dense EN can only be demonstrated by surrogate endpoints, which could not be translated into improved clinical outcomes (morbidity and mortality). Therefore, a large randomized clinical trial that is designed to elucidate the clinical benefits by assessing functional outcomes and infectious complications of calorie-dense EN is required. Moreover, studied patients were not comparable in terms of macro- and micro-nutrients. Thus, it is impossible to determine which component (calories, total protein, glutamine, or micronutrients) had the greatest influence on studied outcomes. However, the aim of our pilot study was not to investigate the effects of a single nutrient. Thus, the present study being a pilot trial, supports further research regarding fluid-restricted and immune-modulated diets. Another limitation is that self-sustained feeding with a regular hospital diet was not considered in daily energy balance, which may have led to an underestimate of actual daily energy provision. However, EN was interrupted amid the recovery of natural meals. Furthermore, patients in the standard group received greater parenteral nutrition when compared to those who received a calorie-dense formula. Whether these differences affected the clinical outcome(s) is currently unknown. In addition, preoperative nutritional screening and assessment also have not been performed.

## 5. Conclusions

Early EN using a calorie-dense and protein rich formula was feasible, leading to an early achievement of calorie and protein targets during the early postoperative course in cardiac surgery patients. Additional studies are encouraged to evaluate the clinical significance of calorie-dense, protein rich EN, which may represent a promising nutrition therapy in cardiac surgery patients.

## Figures and Tables

**Table 1:** Composition of standard and immune formulas for enteral nutrition.

per 100 mL		Standard	Calorie-Dense
Energy	kJ	421	562
	Kcal	100	133
Protein	g	3.80	6.67
*Including glutamine*	g	0.00	2.00
Carbohydrates	g	13.80	18.30
Lipids (total)	g	3.30	3.70
*Saturated fatty acids*	g	0.99	1.80
*(including МСТ)*	g	0.50	1.60
*Essential fatty acids*	g	1.70	0.80
*(including Omega-3)*	g	0.26	0.20
Fiber	g	0.00	1.30
Sodium	mg	100.0	133.00
Potassium	mg	150.0	200.00
Calcium	mg	75.0	99.80
Magnesium	mg	20.0	26.60
Phosphorus	mg	65.0	86.50
Chloride	mg	100.0	133.00
Iron	mg	1.2	1.60
Zink	mg	1.2	2.00
Copper	µg	150.0	200.00
Iodine	µg	13.0	17.30
Chromium	µg	7.0	9.30
Fluorine	mg	0.1	0.13
Manganese	mg	0.2	0.27
Molybdenum	µg	10.0	13.30
Selenium	µg	7.0	12.00
Vitamin А	µg	90.0	120.00
Vitamin D	µg	1.0	1.33
Vitamin Е	mg	1.5	4.00
Vitamin К	µg	7.0	9.30
Vitamin В1	mg	0.2	0.27
Vitamin В2	mg	0.2	0.27
Vitamin В6	mg	0.2	0.27
Vitamin В12	µg	0.3	0.40
Vitamin С	mg	10.0	26.60
Niacin	mg	1.8	2.40
Folic acid	µg	30.0	39.90
Pantothenic acid	mg	0.6	0.80
Biotin	µg	5.0	6.70
Choline	mg	30.0	40.00
Beta-Carotene	mg	0.1	0.13

**Table 2:** Demographic characteristic of studied cohort.

	Standard	Calorie-Dense	*p*-Value
N	20	20	ns
Female	5 (25%)	9 (45%)	ns
Age, years	60.8 (9.3)	61.3 (6.6)	ns
BMI, kg/m^2^	28.3 (5.2)	29.1 (5.5)	ns
Preoperative LVEF, %	58 (16)	53 (18)	ns
Surgery			
CABG	9 (45%)	7 (35%)	ns
HVS	8 (40%)	10 (50%)	
CABG + HVS	2 (10%)	0	
Aortic	1 (5%)	3 (15%)	
Perioperative myocardial infarction	4	5	ns
Rethoracotomy in first 24 h	2 (10%)	2 (10%)	ns
IABP	10 (50%)	10 (50%)	ns
RRT	9 (45%)	8 (40%)	ns
CPB time, min	174 (83)	146 (67)	ns
Aortic cross-clamp, min	66 (28)	49 (27)	ns

BMI = body mass index; LVEF = left ventricle ejection fraction; CABG = coronary artery bypass grafting; HVS = heart valve surgery; IABP = intra-aortic balloon pump; RRT = renal replacement therapy; CPB = cardiopulmonary bypass time; Data presented as mean (SD), or number of patients (%).

**Table 3:** Characteristics of clinical course.

Variable	Group	1 Day	2 Days	3 Days	4 Days	5 Days	6 Days	7 Days	14 Days
Number of survived	Standard	20	20	20	20	20	20	19	18
patients	Calorie-dense	20	20	20	20	20	19	19	19
APACHE II score	Standard	23 (21–25)	23 (20–24)	20 (17–23)	18 (13–23)	16 (13–22)	14 (8–21)	11 (8–24)	7 (7–8)
	Calorie-dense	23 (19–24)	23 (19–24)	21 (16–22)	20 (11–22)	16 (11–21)	12 (9–19)	13 (8–19)	9 (7–14)
SOFA score	Standard	10 (9–12)	10 (8–12)	10 (8–12)	7 (5–10)	6 (4–12)	6 (3–10)	5 (2–10)	2 (0–3)
	Calorie-dense	10 (9–10)	10 (9–12)	9 (8–11)	9 (7–10)	7 (6–9)	6 (4–8)	7 (2–9)	3 (1–8)
VIS	Standard	19 (13–26)	12 (9–15)	7 (2–12)	2 (0–9)	0 (0–4)	0 (0–3)	0 (0–0)	0 (0–0)
	Calorie-dense	17 (9–23)	16 (8–22)	9 (3–16)	5 (0–11)	2 (0–7)	0 (0–2)	0 (0–0)	0 (0–0)
Blood lactate, mmol/L	Standard	5.8 (5.3–8.3)	2.2 (2–2.6)	2 (1.5–2.5)	1.9 (1–2.8)	1.2 (1–2)	1.6 (1.3–5.8)	1.4 (1–8.5)	1.3 (1.1–2)
	Calorie-dense	4.1 (3.8–5.3)	2.7 (2–3.6)	1.9 (1.6–2)	2 (1.5–2.2)	1.6 (1–2.5)	1.4 (1–2.4)	1.8 (1–2.3)	1.4 (1–1.9)
PaO_2_/FiO_2_	Standard	199 (142–240)	175 (136–200)	202 (178–300)	256 (169–343)	234 (190–330)	225 (186–400)	198 (191–379)	
	Calorie-dense	206 (134–253)	171 (125–278)	285 (183–332)	295 (250–394)	359 (278–379)	303 (217–379)	305 (239–374)	

APACHE = Acute Physiology and Chronic Health Evaluation; SOFA = Sequential Organ Failure Assessment; VIS = vasoactive-inotropic score; Data presented as median (25–75 percentile). Hospital mortality was 4 (20%) and 6 (30%) patients in the standard and calorie-dense groups, respectively.

**Table 4:** Characteristics of nutritional support.

Nutritional Provision	Group	1 day	2 day	3 day	4 day	5 day	6 day	7 day
Enteral nutrition, mL/day	Standard	0 (0–0)	500 (462–500)	1000 (650–1250)	1400 (1000–1500)	1000 (350–1500)	1250 (1000–1500)	1250 (1000–1500)
	Calorie-dense	0 (0–0)	500 (500–500)	1000 (500–1500)	1500 (1000–1500)	1500 (1000–1650)	1500 (1250–2000)	1500 (1000–2000)
Enteral nutrition, kcal/day	Standard	0 (0–0)	500 (350–500)	1000 (650–1250)	1400 (1000–1500)	1000 (350–1500)	1250 (1000–1500)	1250 (1000–1500)
	Calorie-dense	0 (0–0)	650 (650–650) *	1300 (650–1950)	1950 (1300–1950)	1950 (1300–2112) **#**	1950 (1625–2600)	1950 (1300–2600) **#**
Nutritional support (EN + PN), kcal/day	Standard	0 (0–0)	500 (350–500)	1000 (650–1200)	2237 (1975–2475)	2475 (2135–2856)	2000 (1625–2000)	1500 (1000–2059)
	Calorie-dense	0 (0–0)	650 (650–650) *	1300 (650–1950)	1950 (1950–2600)	1950 (1950–2600)	1950 (1875–2600)	1950 (1300–2600) **#**
Protein (EN + PN), g/day	Standard	0 (0–0)	19 (17.5–19)	67 (55–73)	93 (66–110)	110 (76–131)	93 (76–110)	57 (38–90)
	Calorie-dense	0 (0–0)	33.3 (33–33) *	67 (33–100)	100 (100–133) #	100 (100–133)	101 (100–133)	100 (67–133) **

EN = enteral nutrition; PN = parenteral nutrition; Data presented as median (25–75 percentiles), * *p* < 0.001, ** *p* < 0.01, # *p* < 0.05 between-group differences (Friedman test).

**Table 5:** Laboratory markers.

Variable	Group	1 Day	3 Days	5 Days	7 Days	14 Days
Prealbumin, g/L	Standard	0.17 ± 0.02	0.12 ± 0.02	0.13 ± 0.03	0.12 ± 0.04	0.13 ± 0.01
	Calorie-dense	0.16 ± 0.02	0.14 ± 0.05	0.15 ± 0.03	0.13 ± 0.04	0.21 ± 0.1 **#**
Transferrin, g/L	Standard	1.61 ± 0.35	1.4 ± 0.31	1.34 ± 0.23	1.46 ± 0.2	1.56 ± 0.55
	Calorie-dense	1.91 ± 0.32	1.79 ± 0.34 **#**	1.85 ± 0.24 #	1.75 ± 0.37 **#**	2.07 ± 0.46
C-reactive protein, mg/L	Standard	5.4 (4.5–8.8)	10 (7.4–19)	9.3 (2.8–14.5)	7.5 (5–8.8)	3.2 (2.1–7.9)
	Calorie-dense	7.4 (5.1–11.2)	14.2 (7.7–20.6)	12 (8.1–12.5)	8.6 (6.1–10.5)	4.5 (2.8–8.6)

Data presented as mean ± SD or median (25–75 percentiles); # *p* < 0.05 between-group differences (mixed-effects model (with group as fixed and time and patient as random effects) with incorporated correction for multiplicity (Tukey’s method)).
